# Autoimmune Diseases and Immune Modulation: Current Research and Future Outlook

**DOI:** 10.3390/biomedicines13122971

**Published:** 2025-12-03

**Authors:** Giuseppe Murdaca, Francesca Paladin, Sebastiano Gangemi

**Affiliations:** 1 Department of Internal Medicine, University of Genova, Viale Benedetto XV, 16132 Genova, Italy; 2 Allergology and Clinical Immunology Unit, San Bartolomeo Hospital, 19038 Sarzana, Italy; 3 Elderly and Disabeld Department, San Paolo Hospital, 17100 Savona, Italy; puell-a@hotmail.it; 4 Allergy and Clinical Immunology Unit, Department of Clinical and Experimental Medicine, University of Messina, Via Consolare Valeria, 98125 Messina, Italy; gangemis@unime.it

Autoimmune diseases (ADs), such as rheumatoid arthritis (RA), Sjogren’s disease (SS), and Systemic Lupus Erythematosus (SLE), have a significant impact on patients’ quality of life and impose significant economic and psychological burdens on society and families. In recent years, the attention has focused on the etiological and pathogenetic mechanisms underlying these immune disorders, with particular reference to the possible mechanisms of immune modulation and potential therapeutic targets. Primary immunodeficiencies, also known as inborn errors of immunity (IEI), certainly represent a fascinating challenge for the research of the mechanisms underlying immune dysregulation. Common variable immunodeficiency (CVID), the most common symptomatic IEI, includes a wide range of clinical manifestations, such as inflammatory and autoimmune diseases, granulomatosis, lymphoproliferation, enteropathy, allergy, and malignancy [1]. It should be remembered that autoimmune manifestations can often precede the diagnosis of CVID. Growing evidence shows that patients with IEI have common genetic defects and pathways that play a role both in the onset of autoimmunity/immune dysregulation and in the risk of developing hematological and solid tumors. This Editorial briefly discusses some of these pathways, with a focus on those already defined in the papers published in the Special Issue. The final aim is to open up the discussion to potential targeted therapies. However, several hypotheses have been proposed to explain how autoimmune disorders can develop in patients with IEI. The research mainly focuses on the maturation and differentiation processes of B-cells that occur in the germinal centers of secondary lymphoid follicles. This complex system is regulated by different populations of lymphoid cells and, in particular, by forkhead box P3 (FOXP3)+ follicular T regulatory cells (Treg) [2]. Tregs, driven by the FOXP3 transcription factor, maintain immune system balance and suppress aggressive immune responses against the body’s own tissues, thus preventing autoimmunity. Mutations in the FOXP3 gene are known to underlie serious autoimmune diseases such as “Immune dysregulation, polyendocrinopathy, enteropathy, and X-linked” (IPEX) syndrome. FOXP3 reduces the synthesis of pro-inflammatory cytokines (i.e., IL-2, IFN-γ, IL-17, TNF-α) by interacting with gene transcription factors (i.e., NFAT, NF-kB, AP-1), and it simultaneously induces the release of immunosuppressive cytokines (i.e., IL-10, TGF-β, IL-35). Furthermore, FOXp3 represses genes typical of CD8+ cytotoxic T lymphocytes, including the perforin gene, granzyme A/B and FasL [3]. Perforin and granulysin levels have been shown to be significantly higher in patients with active SLE, and the proportion of cytotoxic cells (CD8+ granzyme-B+ perforin+ cells) correlates with disease activity. Their integration with traditional biomarkers could improve disease monitoring and management [4]. It goes without saying that these findings confirm how important the control action of FOXP3 is in determining not only the onset of clinical manifestations of SLE but also the degree of activity and severity of the disease. Ex vivo expansion, especially in recurrent ovarian cancer, has been shown to induce an increase in ascitic fluid Tregs. This data confirms that tumor-associated cytotoxic T lymphocyte-related antigen 4 protein CTLA-4+CD25+Foxp3+ Tregs promote tumor cell mass expansion. This could pave the way for the development of adoptive immunotherapy against ovarian cancer [5]. It is hoped that future studies may allow for the adoption of similar immunotherapeutic strategies capable of inducing remission of autoimmune diseases by stimulating the action of FOXP3. Among the molecules that play a central role in the regulation of innate immunity, stimulator of interferon genes (STING) exerts a pivotal action through type I interferon (IFN-I) and pro-inflammatory responses in the presence of cytosolic DNA. STING activation can occur through several pattern recognition receptor (PRR)-dependent and PRR-independent mechanisms including cyclic GMP-AMP synthase (cGAS) protein, interferon gamma inducible protein 16 (IFI16), DEAD-box helicase 41 (DDX41), and DNA-dependent protein kinase (DNA-PK). It is noteworthy that STING activation promotes the infiltration of Foxp3+ Treg cells, confirming the complex network of molecules capable of promoting the onset of autoimmune mechanisms and tumor progression. Confirming the extraordinary complexity of the mechanisms regulating the immune response, it should be considered that signal transducer and activator of transcription 5 (STAT5), activated by various cytokines, including IL2, also represents an important regulator of FOXP3 expression. These signals contribute to the development of autoimmune inflammatory diseases and to the onset and progression of hematological and solid tumors. It should be emphasized that potential immunotherapies selectively modulating STING activity could represent a frontier to be crossed for targeted therapeutic approaches with fewer adverse events. In particular, cGAS-STING inhibitors, although still in their early stages, represent the most credible image of the continuous progress in drug design and targeted translational strategies [6]. Further ahead in the targeted therapeutic challenge are certainly the B-cell-activating factor inhibitors (BAFF-i), which are demonstrating efficacy and safety particularly in patients with both RA and SLE [7]. The importance of vitamin D and microbiome in regulating the immune response must also be emphasized. Simple vitamin D supplementation in cases of proven deficiency and the periodic administration of probiotics can regulate the immune responses and reduce the risk of autoimmunity [8]. However, the application of targeted therapies could represent the key for both the control of autoimmune mechanisms and for restoring immunosurveillance towards malignancy. In particular, allogeneic stem cell transplantation could restore FOXP3+ Tregs, partially controlling immune dysregulation. Gene therapy aimed at restoring the FOXP3 gene in patients’ CD4+ T lymphocytes certainly represents a future that is perhaps not so distant.
